# From Air to Brain: Environmental Nanoparticles as Modifiable Risk Factors for Neurodevelopmental, Neurodegenerative, and Mental Disorders

**DOI:** 10.1021/acsomega.5c12354

**Published:** 2026-06-20

**Authors:** Cristina Hermosillo-Abundis, Oscar Arias-Carrion, Carlos Contreras-Ibáñez, Miguel A. Mendez-Rojas

**Affiliations:** † Department of Chemical-Biological Sciences, Universidad de las Américas Puebla, ExHda. Sta. Catarina Martir s/n, San Andrés Cholula, 72810 Puebla, México; ‡ Experimental Neurology, Instituto Nacional de Rehabilitación Luis Guillermo Ibarra Ibarra Mexico City 14389, Mexico; § Tecnologico de Monterrey, Escuela de Medicina y Ciencias, de la Salud, Mexico City 14380, Mexico; ∥ Department of Sociology, Universidad Autónoma Metropolitana, Unidad Iztapalapa, Av. San Rafael Atlixco 186, Col. Vicentina, México City 09340, México

## Abstract

Ultrafine particles (≤100 nm) and other environmental nanoparticles have emerged as biologically active pollutants that can cross biological barriers, including the blood–brain barrier and the placenta. Growing evidence implicates ultrafine particles in a wide range of neuropsychiatric conditions, yet their effects remain poorly integrated into clinical and public health frameworks. In this review, we distinguish between size-defined ultrafine particles (UFPs, ≤100 nm), composition-defined environmental nanoparticles originating from combustion and secondary formation processes, and engineered nanomaterials (ENPs), which differ in physicochemical properties, exposure scenarios, and regulatory status. This narrative systematic review synthesizes findings from human and experimental studies on the neuropsychiatric and neurodevelopmental effects of environmental nanopollutants. A structured search was conducted in PubMed, Web of Science, Scopus, and Google Scholar up to November 2025, following explicit inclusion and exclusion criteria. Eligible studies included peer-reviewed human and animal research assessing mental health or neurological outcomes of nanopollutant exposure. Epidemiological studies—primarily involving traffic-related air pollution and mixed combustion-derived ultrafine particle exposures—suggest associations with increased risk of cognitive impairment, autism spectrum disorder, depression, schizophrenia, and neurodegenerative diseases, including Alzheimer’s, Parkinson’s, and amyotrophic lateral sclerosis. Prenatal and early life exposures were linked to cortical thinning, altered neurodevelopmental trajectories, and early proteinopathies. Underlying mechanisms include neuroinflammation, oxidative stress, and protein aggregation. Despite methodological heterogeneity, the evidence supports the urgent need for regulation and prevention. Environmental nanopollutants constitute an under-recognized, modifiable risk factor for neuropsychiatric and neurodegenerative conditions. A paradigm shift is needed to incorporate environmental exposure history into mental health research, risk assessment, and prevention strategies. Regulatory action targeting nanopollutant emission and exposure, particularly in vulnerable populations, is critical to mitigating long-term neurological consequences.

## Graphical abstract

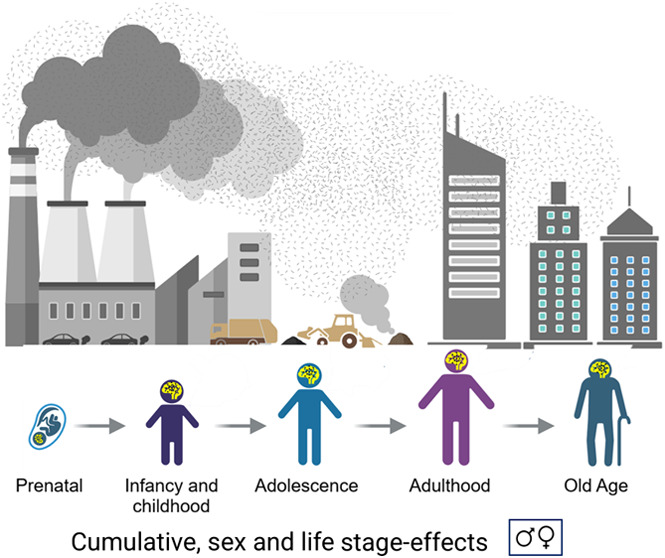

## Introduction

As global industrialization accelerates and urban environments expand, exposure to airborne pollutants has become a critical yet underrecognized threat to neurological health. Among these pollutants, particulate matter (PM)—a complex mixture of solid and liquid particles suspended in air, water, and soil—has attracted increasing concern due to its systemic and neurological effects. Ultrafine particles (UFPs, also referred to as PM_0.1_) are defined as airborne particles with a diameter ≤100 nm. Environmental UFPs exist as complex mixtures comprising carbonaceous cores, adsorbed organic compounds (including polycyclic aromatic hydrocarbons, PAHs), and metals (notably, Fe, Mn, Pb), with composition varying by emission source.^1^ This size range is neurotoxicologically significant for three reasons: (1) enhanced deposition in the alveolar region and olfactory epithelium, (2) ability to translocate across biological barriers through the olfactory nerve, systemic circulation, or bypass the blood-brain barrier (BBB), and (3) high surface area-to-volume ratio enabling greater oxidative potential and cellular reactivity per unit mass compared to larger-particles.^2,3^ Consequently, UFPs constitute a size-based category encompassing chemically heterogeneous particles. Their biological effects depend not only on size but also on source-specific composition (e.g., traffic-derived soot, metal-rich industrial particles, secondary organic aerosols) and copollutant context.

Airborne PM, particularly in urban areas, has been robustly associated with an increased risk of cardiovascular morbidity, including myocardial infarction, stroke, and heart failure.^4,5^ In parallel, respiratory consequences—such as asthma exacerbations, chronic lung disease, and lung cancer—are well documented.^6,7^ Furthermore, exposure to environmental PM is associated with immune dysregulation, increased severity of allergies,^8^ elevated cancer risk,^9^ and a growing body of evidence linking it to autoimmune conditions, including multiple sclerosis and systemic lupus erythematosus.^10–12^

The neurological and psychiatric effects of PM exposure are increasingly evident. Epidemiological and mechanistic studies indicate that exposure to UFPs contributes to the onset and progression of neurodegenerative diseases such as Alzheimer’s disease (AD) and Parkinson’s disease (PD), partly through mechanisms involving neuroinflammation, oxidative stress, and protein aggregation.^13–17^ Associations have also been observed with psychiatric outcomes, including depression, anxiety, and increased risk of suicide,^18–25^ as well as a higher incidence of malignant brain tumors.^26,27^ Cognitive impairments and attention deficits have been documented across age groups,^28,29^ while acute and chronic PM exposure is recognized as a modifiable risk factor for ischemic stroke.^30–32^

Children appear especially vulnerable. Prenatal and early life exposure to PM has been associated with neurodevelopment, brain morphology, and cognitive performance alterations, with potential long-term consequences for social behavior and mental health.^15,33–36^ However, much of the existing literature has focused on larger particle fractions, particularly PM_2.5_, or has relied heavily on preclinical models, leaving important gaps in our understanding of the specific risks posed by UFPs and engineered nanoparticles (ENPs) to the human brain.^37,38^ A schematic conceptual framework, linking particle characteristics to neurological outcomes, is shown in Figure 1.

**Figure 1:**
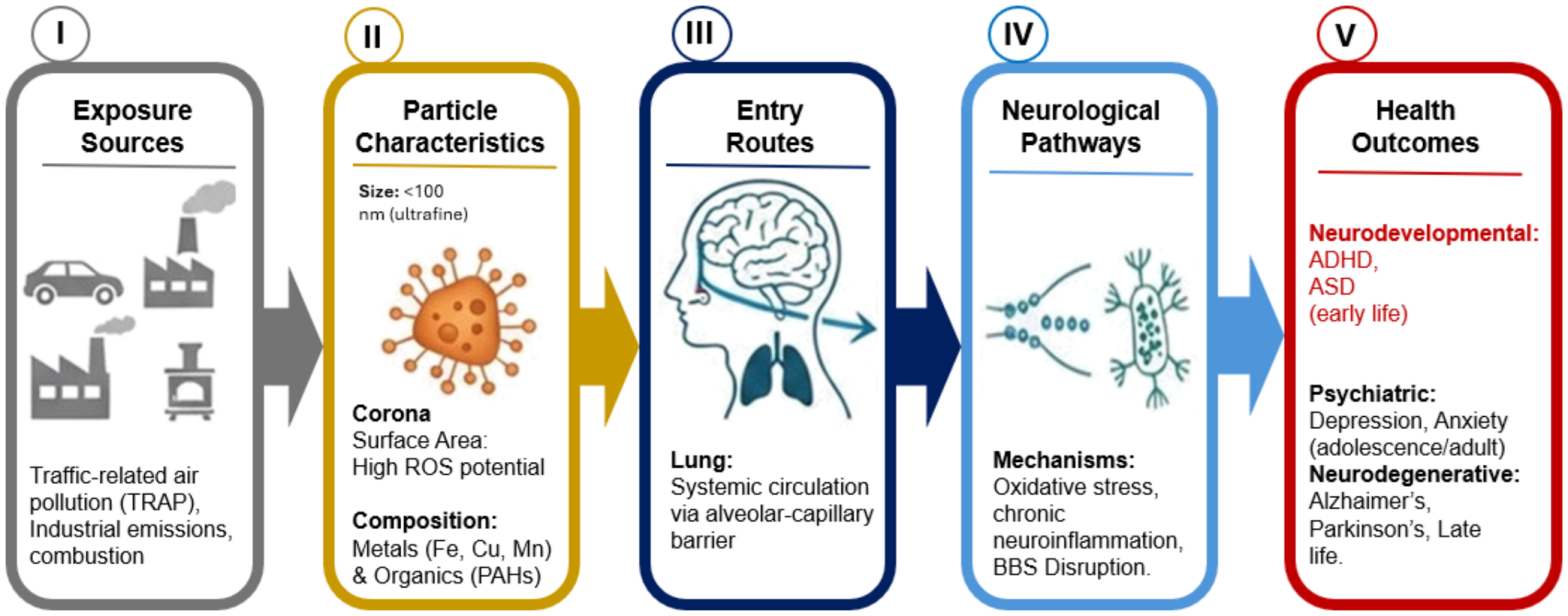
Conceptual framework linking UFP exposure to neurological health outcomes across the lifespan. *(I) Exposure Sources:* Traffic-related air pollution (TRAP), industrial emissions, and combustion processes constitute the primary urban UFP burden. *(II) Particle Characteristics:* UFPs (<100 nm) exhibit a high surface area-to-volume ratio, elevated ROS generation potential, and complex chemical composition including redox-active metals (Fe, Cu, Mn) and polycyclic aromatic hydrocarbons (PAHs). *(III) Entry Routes:* Inhaled UFPs translocate to systemic circulation via the alveolar-capillary barrier and directly to the CNS via the olfactory pathway. *(IV) Neurological Pathways:* Key neurotoxic mechanisms include oxidative stress, neuroinflammation, and disruption of the BBB, driving microglial activation and neuronal damage. *(V) Health Outcomes:* Neurological outcomes stratified by life stage: neurodevelopmental disorders (ADHD, ASD) following early life exposure; psychiatric disorders (depression, anxiety) across adolescence and adulthood; and neurodegenerative conditions (Alzheimer’s and Parkinson’s disease) linked to cumulative late-life exposures. Arrows indicate the direction of progression from environmental exposure to clinical outcome.

A recent review of serial cohort studies shows that the effects of UFPs extend into young adulthood and adulthood, accelerating cognitive decline and increasing the risk of early dementia, and their presence is associated with reports of mood disorders such as depression and anxiety in diverse populations.^39^ These authors point out that chronic exposure to particles reaches the nervous system via a systemic or olfactory route, triggering the production of reactive oxygen species (ROS) and activation of pro-inflammatory pathways (e.g., nuclear factor-kappa B, NF-κB), overactivation of microglia/astrocytes, disruption of the blood-brain barrier (BBB), mitochondrial damage, and epigenetic alterations. These responses, when persistent, induce synaptic dysfunction, loss/alteration of neurogenesis, and cerebrovascular injury, which would explain the cognitive decline in the more exposed populations. In parallel, systemic inflammation and oxidative stress are plausible mechanisms for disrupting monoamine neurotransmission and neuroendocrine regulation (e.g., hypothalamic-pituitary adrenal, HPA, axis), which could mediate depressive and anxiety symptoms. Hence, this revision remains pertinent to mental health, both in its cognitive and affective instances.

Current air quality regulations are primarily based on mass concentration metrics (PM_2.5_ and PM_10_) established through frameworks such as the US EPA’s Integrated Science Assessment for Particulate Matter and the WHO Air Quality Guidelines.^40,41^ The US EPA risk assessment framework employs a weight-of-evidence approach integrating toxicological, epidemiological, and exposure data to establish National Ambient Air Quality Standards (NAAQS), while WHO guidelines provide globally applicable recommendations linking specific pollutant concentrations to health outcomes. However, both frameworks acknowledge significant gaps in addressing UFPs. The US EPA’s 2019 Integrated Science Assessment concluded that evidence for UFP health effects was “suggestive but not sufficient” for establishing separate standards, citing challenges in exposure assessment, lack of standardized measurement protocols, and difficulty disentangling UFP effects from copollutants.^40^ Similarly, the WHO 2021 Air Quality Guidelines recognized emerging evidence for UFP toxicity but stopped short of recommending numeric targets, instead calling for further research on particle number concentration (PNC) metrics and source-specific health impacts.^41^

This review addresses this regulatory gap by synthesizing recent evidence specifically linking ultrafine particle exposure to mental health and neurodevelopmental outcomes—end points that are not comprehensively addressed in existing PM mass-based frameworks. Rather than proposing an entirely novel paradigm, we argue for evolution and expansion of current frameworks to incorporate: (a) Particle number concentration (PNC) metrics alongside mass-based standards, as PNC better captures UFP exposure; (b) Source-specific regulations targeting high UFP-emitting sources (traffic, industrial, cooking emissions); (c) Explicit consideration of neuropsychiatric end points in health impact assessments, particularly for vulnerable populations (children, elderly, individuals with pre-existing mental health conditions). This approach builds upon, rather than replaces, established EPA and WHO frameworks by addressing recognized evidence gaps and incorporating emerging science on nanoparticle neurotoxicity. The “paradigm shift” we advocate is not in the fundamental risk assessment methodology—which remains sound—but in the exposure metrics, health end points, and vulnerable populations prioritized within these existing frameworks. Also, this systematic review addresses other critical knowledge gaps by gathering fragmented evidence across disorder categories into a common place, pointing out the incomplete information characterization of UFPs and the disconnection with their mechanism-outcome, and the identification of vulnerable population, by synthesizing the available evidence on the effects of UFPs on mental health and brain function in children and adults, but limiting causal inference. Our contribution is 2-fold: first, we apply a systematic review design with explicit inclusion and exclusion criteria; and second, we adopt a life-course vulnerability framework that integrates epidemiological, mechanistic, and neuropathological evidence across developmental, adult, and aging stages. By considering nanopollutants as modifiable risk factors, this review underscores their translational relevance for clinical practice and public health. A clearer understanding of these risks is essential to inform prevention strategies, guide regulatory action, and reshape approaches to diagnosing and managing pollution-associated brain disorders.

## Methods

### Eligibility Criteria

We included original, peer-reviewed research articles published in English that investigated the association between exposure to environmental nanoparticles, including UFPs and nanopollutants, and mental health or neuropsychiatric outcomes across all stages of neurodevelopment. We excluded reviews, meta-analyses, editorials, book chapters, conference abstracts, and nonpeer-reviewed literature. Studies based on animal or in vitro models were included if they reported neurobiological, behavioral, or molecular outcomes relevant to human mental health or neurodevelopmental disorders.

### Information Sources

We searched four electronic databases: PubMed/MEDLINE, Web of Science, Scopus, and Google Scholar, from January 1, 2000 to November 30, 2025. The start date of 2000 was selected because standardized UFPs measurement techniques (condensation particle counters, scanning mobility particle sizers) became widely available in environmental research around 2000; aside from that, the concept of UFPs as a distinct health-relevant exposure metric emerged in the late 1990s-early 2000s, and also because earlier studies lacked adequate exposure characterization to distinguish UFP effects from general particulate matter. While a few foundational studies from the 2000–2009 were included, others from 2008 to 2014 were selected as they established the field when UFP measurement technologies became standardized, and the paradigm shift toward measuring particle number concentration and direct brain translocation studies emerged;^42^ finally, the bulk of systematic evidence appeared in the 2015–2025 period, indicating a rapid expansion of the field. Reference lists of eligible articles and relevant reviews were manually screened to ensure comprehensive coverage.

### Search Strategy

Search terms included Medical Subject Headings (MeSH) and free-text terms related to environmental nanoparticle exposure and mental health outcomes. Key terms included: “environmental nanoparticles”, “ultrafine particles”, “UFP”, “nanopollutants”, “nanoparticles”, “nanomaterial”, “particle number concentration”, “airborne nanoparticle”, “PM0.1”, “mental disorders”, “mental health”, “depression”, “depressive disorder”, “anxiety”, “anxiety disorder”, “mood disorder”, “affective disorder”, “psychological distress”, “psychiatric”, “neuropsychiatric”, “neurodevelopment”, “developmental delay”, “intellectual disability”, “learning disorder”, “developmental disease”, “autism”, “autism spectr disorder”, “ASD”, “attention deficit”, “attencion deficit hyperactivity disorder”, “ADHD”, “cognitive function”, “cognitive decline”, “cognitive impairment”, “mild cognitive impairment”, “MCI”, “dementia”, “Parkinson disease”, “Alzheimer”, “neurological”, “neurotoxicity”, “neurogedegeneration”, “neuroinflammation”, “neural”, “neuronal”, “glial activation”, “microglia”, “astrocyte”, “neurotransmitter”, “blood-brain barrier”, “BBB disruption”, “oxidative stress”, “dopamine”, “serotonin”, and “brain”. Boolean operators (AND, OR) were used to combine terms.

### Selection Process

Two independent reviewers screened titles and abstracts against the eligibility criteria. Full texts were retrieved for articles deemed potentially eligible. Discrepancies were resolved through discussion and, when necessary, adjudicated by a third reviewer. The selection process followed PRISMA 2020 flow diagram guidelines. No previous protocol was registered, and no quantitative meta-analysis or statistical review was intended, but we take the hitherto described measures and decisions to refrain from bias or exclusions.

### Data Collection Process

Data extraction was performed independently by two reviewers using a standardized data extraction form. Extracted data included: (a) Study characteristics (author, publication year, country, study design, population, sample size); (b) Exposure characteristics (type and source of nanoparticles, exposure route, duration, and setting); (c) Exposure characterization (measurement method, particle size range, concentration levels, copollutants); (d) Mental health outcomes (diagnostic, behavioral, and cognitive assessments); (e) Neurological end points (structural or functional brain changes, developmental or degenerative markers); (f) Biomolecular outcomes (oxidative stress markers, neuroinflammatory signals, protein misfolding, and gene expression profiles); (g) Additional variables, such as participant age, sex, socioeconomic status, and comorbidities, when available. Any disagreements in data extraction were resolved through consensus or consultation with a third reviewer.

## Results and Discussion

### Nanopollutants: Classification and Neurologically Relevant Sources

The increasing ubiquity of nanomaterials in the environment has raised growing concerns regarding their neuropsychiatric impacts. Environmental nanoparticles —typically less than 100 nm in diameter—undergo complex physicochemical transformations that affect their biological behavior and neurotoxic potential. Key physicochemical determinants of neurotoxicity include particle size, surface chemistry, chemical composition, agglomeration state, and morphology.^43^ Environmental UFPs originate from three primary categories: traffic and vehicular emissions, industrial and indoor combustion, and natural or engineered sources.^44–49^ Of these, combustion processes represent the dominant pathway for human neurological exposure, given their quantitative dominance in urban air and their generation of compositionally complex particles with high oxidative potential.

### Airborne Sources of Nanopollutants: Prioritizing Neurologically Relevant Exposures

Traffic emissions constitute the dominant urban UFP source by particle number concentration, accounting for 60% on average and up to 90% near heavily trafficked roadways—substantially exceeding road traffic’s contribution to PM mass.^50^ Sub-50 nm particles originating from diesel exhaust are of particular neurological concern due to their high lung deposition efficiency, deep respiratory tract penetration, and potentially harmful chemical composition including redox-active metals and PAHs.^51^ Beyond exhaust, nonexhaust sources—brake and tire wear and resuspended road dust—contribute additional nanoparticle loads with variable metal composition relevant to neurotoxicity.^52^

Indoor combustion represents a particularly significant yet under-recognized exposure pathway. During gas cooking, indoor nanocluster aerosol (NCA) emission factors can reach approximately 10^16^ particles/kg-fuel, exceeding those from gasoline- and diesel-powered vehicles. A single 20 min cooking event typically releases between 10^13^ and 10^16^ nanoclusters.^53^ Solid fuel combustion generates UFP emissions approximately one to 3 orders of magnitude higher than liquid fuels or gas.^54^ After just 15 min of cooking, over 90% of emitted particles fall within the ultrafine range, with inhalation constituting the primary exposure route.^55^ These indoor exposure scenarios are particularly relevant given the prolonged duration of exposure and the vulnerability of individuals—including children and pregnant women—in domestic settings.

Natural sources (volcanic eruptions, forest fires, soil erosion) contribute to baseline nanoparticle concentrations. However, they are substantially lower than anthropogenic combustion sources in urban environments and are not further addressed in this review, given its focus on modifiable exposure risks.^44–46^

### Exposure Routes to the Brain: From Inhalation to Neurological Effects

Inhalation is the primary route by which UFPs reach the central nervous system, through two well-characterized pathways:

#### (1) Olfactory Pathway (Nose-to-Brain)

This route directly bypasses the blood-brain barrier (BBB). Following nasal deposition, UFPs are taken up by olfactory receptors and transported retrogradely along olfactory axons to the olfactory bulb and higher brain structures.^56–58^ Neuronal transport velocity along olfactory axons has been calculated at approximately 2.5 mm/hour, encompassing deposition on olfactory mucosa, receptor uptake, retrograde translocation within olfactory dendrites, and anterograde movement into the olfactory bulb.^59^ Approximately 16% of 20 nm particles translocate from the olfactory region to the olfactory bulb immediately after exposure, demonstrating that particle size critically determines translocation efficiency and timing.^60^

#### (2) Pulmonary-Systemic Pathway

Alveolar deposition of UFPs is followed by translocation into the systemic circulation and subsequent BBB crossing. While the healthy BBB provides partial protection, pathological conditions, including hypertension, neuroinflammation, and pollution-induced barrier disruption, increase permeability to circulating UFPs.^61^ Once in the brain via either route, UFPs trigger neuroinflammation, oxidative stress, and proteinopathy cascades relevant to both psychiatric and neurodegenerative outcomes.^62,63^

Particle size governs deposition site and translocation efficiency: nanoparticles smaller than 10 nm deposit predominantly in the alveoli, while those larger than 10 nm deposit more in the nasal cavity.^64^ This size-dependent deposition has direct implications for predicting neurological risk from different UFP sources.

Compelling evidence from urban pediatric populations confirms that children in polluted environments show neuroinflammation, neurodegeneration, and cognitive developmental delays consistent with these mechanistic pathways.^63^ The convergence of high-level urban UFP exposure with direct olfactory access to the brain—bypassing conventional BBB protections—constitutes the central exposure scenario motivating this review’s focus on neurological and mental health outcomes. It is essential to emphasize that exposure levels and physicochemical controls in nanopharmaceutical applications differ substantially from those in incidental environmental exposures. Therefore, extrapolation between these contexts should be interpreted cautiously.

Figure 2 summarizes the sources, exposure pathways, and brain effects of environmental nanoparticles, emphasizing inhalation as the dominant entry route, while also considering other environmentally relevant exposure pathways.

**Figure 2:**
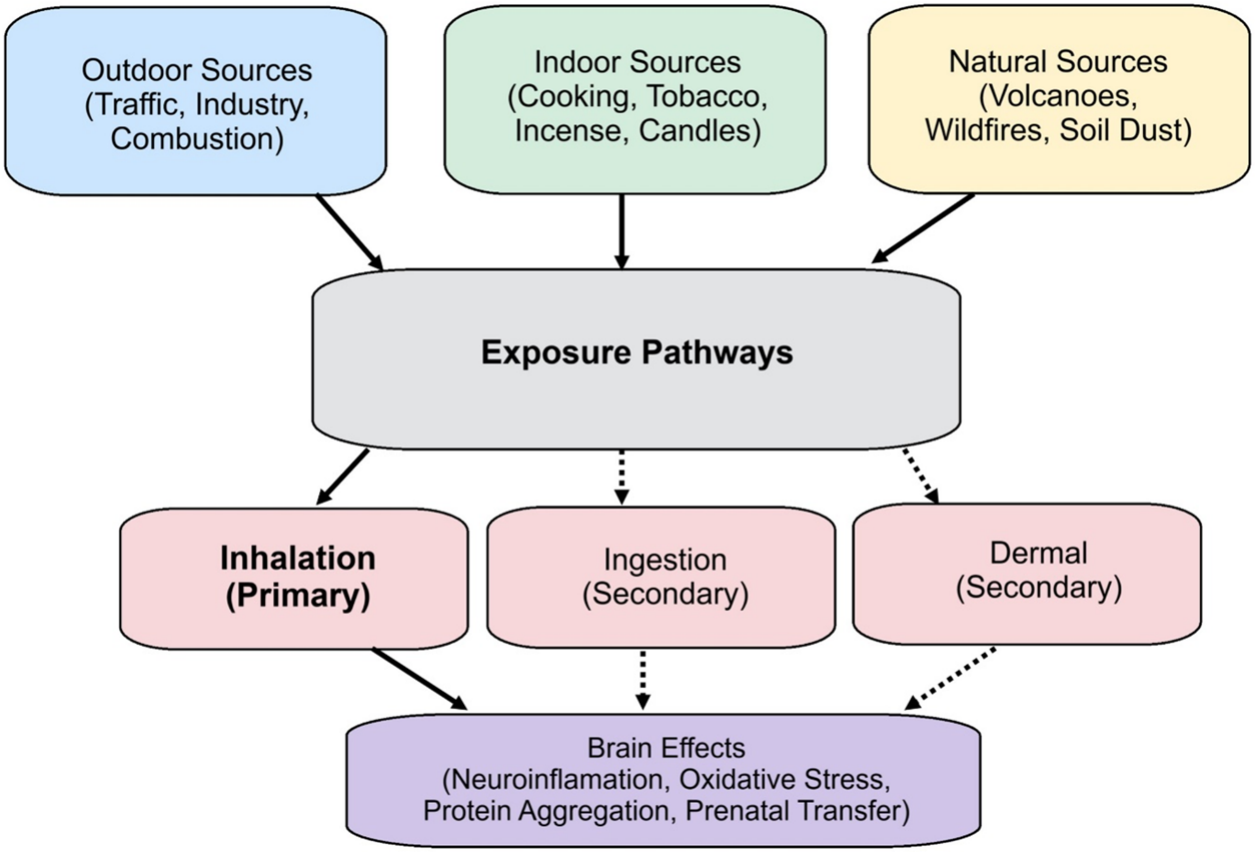
Sources, exposure pathways, and brain effects of environmental nanoparticles. Environmental nanoparticles originate from outdoor sources (traffic, industrial emissions, combustion), indoor sources (cooking, tobacco smoke, incense, candles), and natural sources (volcanic eruptions, wildfires, soil dust). **Inhalation** is the predominant exposure pathway, enabling nanoparticles to enter the respiratory tract and reach the brain via systemic circulation or direct olfactory nerve transport. **Ingestion** (through food, water, or swallowed particles) and **dermal absorption** represent secondary pathways. Once internalized, nanoparticles can cross biological barriers, including the blood–brain barrier and the placenta, leading to neuroinflammation, oxidative stress, protein aggregation, and potential impacts on neurodevelopment and neurodegeneration.

The historic evolution of scientific evidence linking air pollution, UFPs, and neurological and mental health outcomes has been dynamic since 1990s to the date. Figure 3a schematically presents a visual timeline illustrating how this topic has evolved in time along four distinct research eras characterized by progressive methodological refinement and conceptual expansion: **(i) Foundational Studies (1990s–2000s)** recognizing that air pollution affects the brain through epidemiological observations linking PM_10_/PM_2.5_ to cognitive decline and residential proximity to major roadways, neuropathological findings demonstrating neuroinflammation markers and amyloid-β plaques in exposed children with particulate matter detected in olfactory bulb and frontal cortex, and toxicological evidence showing diesel particle-induced microglial activation and oxidative stress. Critical limitation: inability to distinguish ultrafine particle effects from larger PM fractions. **(ii) UFP-Focused Research (2008–2015)** where a paradigm shift enabled by technological advancement through widespread availability of condensation particle counters (CPCs) and scanning mobility particle sizers (SMPS) for particle number concentration and size distribution measurement occurred; key mechanistic discoveries included direct brain translocation within 4 h via olfactory pathway bypassing the blood-brain barrier, and size-dependent toxicity demonstrating 14 nm particles produced greater neurotoxicity than 56 nm particles. Initial epidemiological evidence using a UFP tracer linked exposure to cognitive decline and behavioral problems in children, with socioeconomic status identified as vulnerability factor. Gap: reliance on black carbon proxy rather than direct PNC measurement; mental health outcomes not systematically assessed. **(iii) Mental Health Focus (2015–2020)** characterized by an explosive growth in UFP-mental health research with pan-neurological recognition across neurodevelopmental (ASD, ADHD), psychiatric (depression, anxiety), and neurodegenerative (dementia) disorders. Major findings from multicountry cohorts (US, Sweden, UK, Italy, Canada) demonstrated PM_2.5_ associated with 27% increased dementia admissions (13.6 million participants), traffic-related NO_2_ linked to 9% increased autism risk, and PM_2.5_ correlated with 72% increased odds of psychotic experiences. Limitations: continued reliance on PM_2.5_/NO_2_ proxies rather than direct UFP measurement; predominance of cross-sectional study designs. **(iv) Mechanistic Integration (2020–2025)** a period of methodological maturation characterized by four key advances: direct particle number concentration measurement via urban monitoring networks demonstrating PNC associations independent of PM_2.5_ with psychiatric emergency department visits; source apportionment studies distinguishing traffic versus cooking versus industrial UFP sources and identifying source-specific neurotoxicity patterns; human biomarker studies using brain MRI to demonstrate white matter microstructural changes and plasma inflammatory markers (IL-6, TNF-α) elevation correlating with depressive symptoms; and longitudinal psychiatric study designs with 7-year follow-up periods (N = 88,746) predicting incident depression while controlling for baseline mental health status. Achievement: unified pathway model integrating exposure-mechanism-outcome relationships across the neurological disease spectrum. The timeline demonstrates the field’s evolution from foundational recognition of air pollution’s neurotoxicity to a mechanistically grounded, exposure-specific understanding of ultrafine particle impacts on mental health and neurological function across the lifespan. Figure 3b visualizes the cumulative growth of publications across five research categories: *Neurodevelopmental Disorders*, *Psychiatric/Mood Disorders*, *Neurodegenerative Disorders*, *Mechanistic Studies*, and *Review Articles*. Shaded background regions demarcate the eras. The analysis demonstrates exponential research growth from 3 publications during the foundational era to 78 publications in the current era (2020–2025), with mechanistic studies constituting the largest proportion (34.3%), followed by psychiatric/mood disorders (23.1%), neurodegenerative disorders (22.4%), and neurodevelopmental disorders (15.4%), reflecting increased recognition of air pollution as a pan-neurological risk factor with particular emphasis on mental health outcomes. Notable trends include the emergence of psychiatric/mood disorder research post-2015, accounting for 26% of publications in the current era, and the continued mechanistic research emphasis (32%) supporting translational understanding of exposure-outcome pathways. The temporal distribution reflects the field’s maturation from sparse foundational work establishing air pollution’s neurological effects to a robust, multidisciplinary research area integrating epidemiological, clinical, and mechanistic evidence across the neurological disease spectrum.

**Figure 3:**
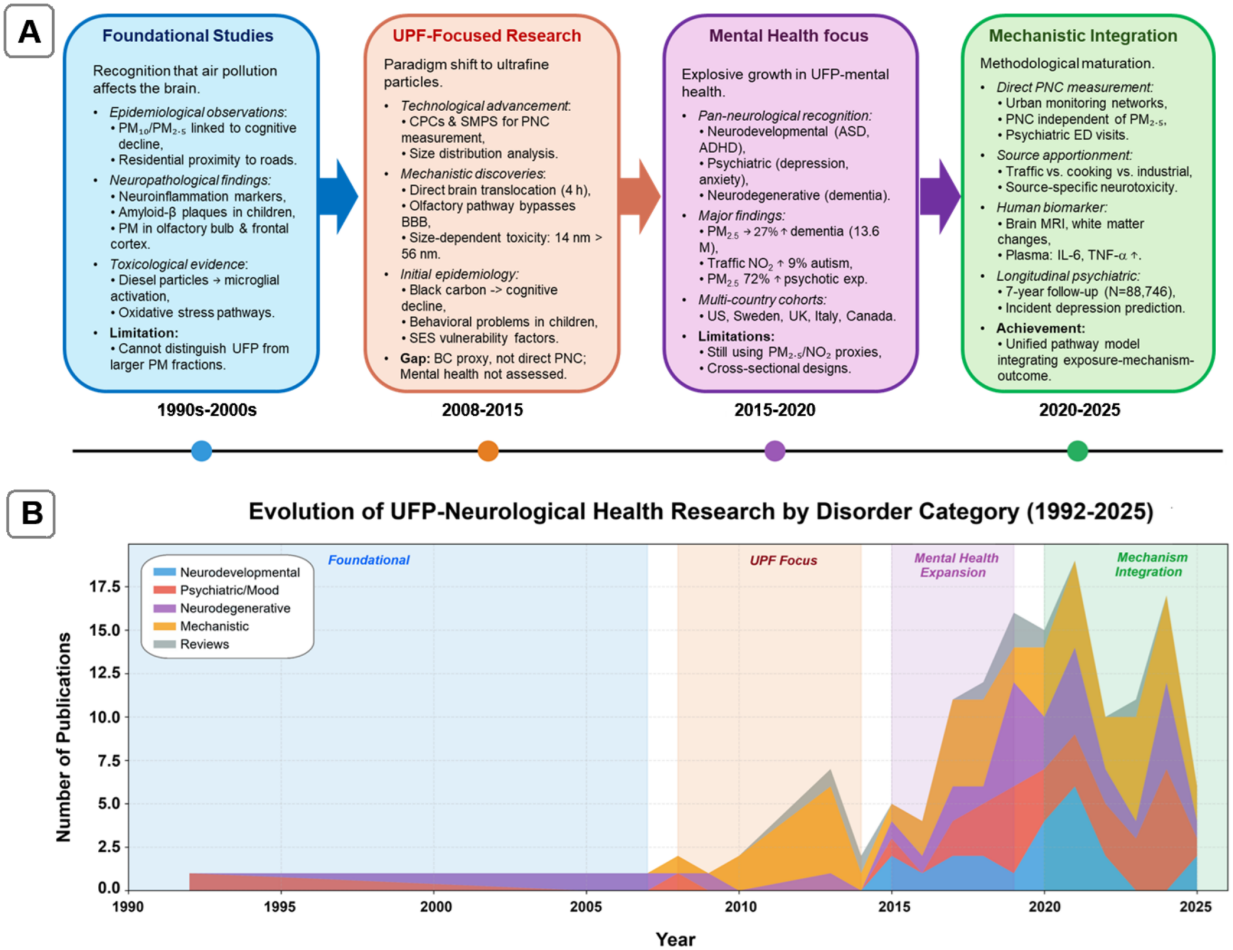
(A) Evolution of research on UFPs and neurological health (1990s–2025). (B) Stacked area chart showing the temporal distribution of research publications on UFPs and neurological health by disorder category (1992–2025) across five categories: *Neurodevelopmental* (blue), *Psychiatric/Mood* (red), *Neurodegenerative* (purple), *Mechanistic* (orange), and *Reviews* (gray). Shaded backgrounds indicate corresponding research eras: *Foundational* (1990–2007), *UFP Focus* (2008–2014), *Mental Health Expansion* (2015–2019), and *Mechanistic Integration* (2020–2025).

### Etiology and Neuropathological Pathways of Mental Illness

Neurodevelopmental disorders constitute complex, heterogeneous conditions emerging from the interplay of biological, psychological, and social factors. Clinically, these disorders encompass disturbances in cognition, emotion, and behavior ranging from transient stress-related reactions to chronic, disabling syndromes such as schizophrenia, bipolar disorder, major depressive disorder, and neurodevelopmental conditions including attention-deficit/hyperactivity disorder (ADHD) and autism spectrum disorder (ASD).^65,66^ Diagnostic boundaries frequently overlap, with substantial symptom co-occurrence complicating clinical classification and treatment. To organize the mechanistic insights, we present information organized into three thematic pathways: neurodevelopmental disorders from early life exposures, psychiatric/mood disorders across the lifespan, and neurodegenerative disorders as late-life outcomes.

#### (i) Neurodevelopmental Disorders (Early Life Exposures)

Neurodevelopmental disorders manifest during childhood and adolescence, involving impaired brain development. This category includes autism spectrum disorder (ASD), attention-deficit/hyperactivity disorder (ADHD), intellectual disability, and developmental delays. Epidemiological studies demonstrate moderate associations between prenatal and early childhood UFPs exposure and neurodevelopmental outcomes. A systematic review and meta-analysis by Lam et al. (2016) reported pooled odds ratios of 1.07 (95% CI: 1.03–1.12) per 10 μg/m^3^ increase in PM_2.5_ for ASD.^67^ More recent studies using direct UFPs measurements show stronger associations: Alemany et al. (2018) found traffic-related UFPs exposure during pregnancy associated with ASD (OR = 1.86, 95% CI: 1.21–2.85 for highest vs lowest quartile).^68^ For ADHD, a Swedish nationwide cohort (N = 508,010) by Markevych et al. (2018) demonstrated associations with traffic-related air pollution (HR = 1.09, 95% CI: 1.04–1.15).^69^ More specifically, Oudin et al. (2019) reported prenatal NO_2_ exposure (UFPs proxy) associated with increased ADHD risk (OR = 1.06 per 10 μg/m^3^, 95% CI: 1.01–1.12).^70^ A critical developmental window has been identified.

##### Critical Developmental Window and Exposure Effects

The prenatal period, particularly the second and third trimesters, shows the strongest associations with neurodevelopmental outcomes. Volk et al. (2013) demonstrated that third-trimester traffic-related air pollution exposure was most strongly associated with ASD (OR = 2.12, 95% CI: 1.06–4.24).^71^ Early postnatal exposure (first year of life) also shows significance: Chen et al. (2018) found that PM_1_ exposure during the first year of life was associated with ASD in a Chinese case-control study (OR = 1.68, 95% CI: 1.32–2.14).^72^ A recent study by Morrel et al. (2025) confirmed that timing matters: exposures during neurogenesis and synaptogenesis periods (weeks 8–24 of gestation) showed the strongest associations with neurodevelopment and potential cognitive deficits.^73^

The TLR4/NF-κB → Microglial Activation → Synaptic Pruning Pathway is particularly critical during neurodevelopment. Maternal LPS treatment results in significant reduction in synaptic pruning-related proteins C3 and CR3A levels, with TLR4 signaling pathways protein expression levels—including TLR4, Phospho-NFκB p65, IKKα, and IBA-1, and iNOS—increased in LPS-treated offspring, whereas Arg-1 was decreased. Pregnancy LPS treatment induces ASD-like behavior in adult offspring requiring TLR4 signaling pathway activation of prefrontal cortex microglia, with abnormally activated microglia interfering with neural circuits through synaptic structure (dendritic spine) phagocytosis and clearance, affecting synaptic plasticity and leading to neurodegenerative disease.^74^

##### Complement-Mediated Synaptic Eimination in Development

During normal development, high microglial CR3 expression recognizes immature neuronal C3 receptors and prunes neuronal dendrites to maintain normal neuronal connections, whereas abnormal microglial activation may disrupt synaptic pruning, resulting in neurological dysfunction.^74^ C1q and downstream C3 are implicated in synaptic pruning—a process wherein synapse subsets are eliminated while remaining synapses are preserved and strengthened. C1q and C3 are not only widely expressed in the CNS but also localize to immature synapse subsets in the developing visual system, leading to these “tagged” axons and synapse elimination.^75^

Microglia mediate synaptic pruning via phagocytosis, recognizing complement-tagged synapses using complement receptors, including CR3. Mice lacking C3 or CR3 show reduced synaptic pruning, indicating that deficient complement signaling during brain development results in long-term synaptic connectivity defects.^76^ During early postnatal development, astrocytes induce neuronal C1q expression through TGF-β, and C1q colocalizes with synapses. Complement protein C3, which also colocalizes with synaptic puncta, is enzymatically cleaved to smaller fragments C3a and C3b, with microglia engulfing synapses through iC3b (the C3b cleavage product) interaction with its CR3 receptors.^77^

##### Pathological Synaptic Pruning in ASD

Synaptic pruning downregulation during development potentially contributes to cortical hyperconnectivity and behavioral symptoms characterizing individuals with autism, epilepsy, and schizophrenia.^77^ In psychiatric disorders, complement components C1q, C4, and C3 fragment iC3b tag inactive synapses during postnatal development. C1q-, C3-, and C4-deficient mice display higher excitatory synapse density and abnormalities in neural circuit development. C1q-deficient mouse models display decreased activated glia levels, and aged C3-deficient mice show reduced age-dependent synapse and neuronal loss in hippocampal CA3, together with enhanced long-term potentiation and cognition.^78^

##### Early Life Brain Morphological Changes

Emerging neuroimaging and histopathological evidence suggest that early life UFPs exposure associates with measurable brain morphological changes. In a Dutch population-based study, higher ambient nanopollutant exposure was linked to reduced cortical thickness in the right postcentral gyrus—a region implicated in sensory integration, attention, and psychiatric vulnerability.^79,80^ Although conducted in a relatively low-exposure environment, these findings support concerns that even moderate levels of pollution may influence brain development.

##### Early Life Proteinopathy and Long-Term Risk

Striking histopathological evidence from urban pediatric autopsies in Mexico City revealed dopaminergic neuronal degranulation in the substantia nigra of children as young as toddlers, alongside early neurodegenerative changes including hyperphosphorylated tau, β-amyloid, α-synuclein, and TDP-43.^81^ These proteinopathies, typically associated with adult neurodegenerative diseases, raise possibilities that early nanopollutant exposure may confer lifelong vulnerability to both neurodegenerative and psychiatric disorders through cumulative dopaminergic system damage.

##### Epidemiological Evidence—Cognitive and Behavioral Consequences

A Shanghai cohort study found early life PM_1_ and PM_2.5_ exposure—both including ultrafine fractions—significantly increased ASD risk, with the second and third life years emerging as critical vulnerability periods.^72^ The Barcelona-based BREATHE project reported similar concerns: children attending schools with high traffic-related air pollution levels demonstrated significantly reduced cognitive development, particularly in working memory and attention, over multiple years.^82,83^ Notably, although elemental carbon and UFPs were measured separately in these studies, the typical particle size falls within the ultrafine range, underscoring the need for improved nanopollutant characterization in future research.

##### Metal-Specific Neurodevelopmental Effects

Potter et al. (2021) reported epidemiological and toxicological evidence of fine (PM_2.5_) and ultrafine (PM_0_._1_) particle exposure effects and associated metals (such as Mn, Pb, Cu, Zn, Ni, Ba) on neurotoxicity and mental health.^84^ They find evidence that PM-bound Mn associates with impaired infant cognitive development, Mn and Cu with basal ganglia structural alterations and decreased motor performance and IQ deficits in schoolchildren (observed in the BREATHE cohort study), and Mn with impaired motor skills in older adults.

##### Early Exposure Consequences for Adult Mental Health

Further reviews have identified cognitive impairments following early exposure to air pollution during pregnancy and childhood, including inattention, hyperactivity, and altered brain architecture. These effects are thought to be mediated by oxidative stress, neuroinflammation, and endocrine disruption, though the precise mechanisms remain incompletely elucidated.^85^ Growing evidence suggests links between air pollution exposure (PM_2.5_ and NO_2_) and increased risk of developing ASD or ADHD, possibly through mechanisms like neurotoxicity and inflammation.^70,85^

##### Developmental HPA Axis Vulnerabilities

In overweight and obese Latino youth, higher ozone exposure in the prior 1–7 months was associated with higher serum morning cortisol, while longer-term PM_2.5_ exposure (4–10 months) was associated with lower serum morning cortisol levels; pubertal status stratification showed associations in prepubertal children compared to pubertal and postpubertal children.^86^ These findings suggest nanopollutants produce temporal and developmental stage-specific HPA axis alterations.

UFPs exposure during critical developmental windows disrupts neurogenesis, synaptogenesis, and myelination. Mechanistic studies demonstrate that nanoparticles induce oxidative stress and neuroinflammation during brain development, thereby altering neuronal migration and synaptic pruning. Block and Calderón-Garcidueñas (2009) review evidence for microglial activation triggering excessive synaptic elimination via dysregulation of the complement cascade.^87^ Allen et al. (2017) demonstrated in animal models that developmental UFPs exposure impairs hippocampal neurogenesis and alters dopaminergic neurotransmission, consistent with ADHD pathophysiology.^28^ Recent work by Fathian-Nasab et al. (2025) showed that prenatal nanoparticle exposure disrupts blood-brain barrier maturation, leading to persistent neuroinflammatory signaling.^88^ White matter microstructure abnormalities, measured by diffusion tensor imaging, have been documented in children with higher UFPs exposure, suggesting myelination disruption.^79^

There are some key limitations to these studies; for example, lower socioeconomic status correlates with both higher pollution exposure and increased diagnostic rates for neurodevelopmental disorders, potentially inflating associations, although most studies cannot clearly separate UFPs effects from correlated pollutants (NO_2_, PAHs, PM_2.5_). Therefore, ASD and ADHD diagnostic criteria evolved, and diagnostic ascertainment varies by healthcare access, resulting in residential-address-based models that may not capture personal exposure, especially among mobile populations.

#### (ii) Psychiatric/Mood Disorders (Across Lifespan)

Adult-onset or recurrent mood and anxiety disorders, including major depressive disorder (MDD), generalized anxiety disorder (GAD), bipolar disorder, and related psychiatric conditions affecting individuals across the lifespan. The epidemiological evidence shows weak to moderate associations between UFPs exposure and psychiatric disorders. A systematic review and meta-analysis by Braithwaite et al, including 25 studies, found pooled effect estimates of OR = 1.10 (95% CI: 1.04–1.17) per 10 μg/m^3^ PM_2.5_ for depression.^18^ Studies using more specific UFPs metrics show stronger associations: Yang et al. reported that long-term exposure to multiple air pollutants, including UFPs, associated with incident depression (HR = 1.16, 95% CI: 1.10–1.23) and anxiety (HR = 1.11, 95% CI: 1.06–1.18) in a UK Biobank cohort (N = 389,185).^26^ Time-series studies demonstrate short-term associations: Qiu et al. found that same-day PM_2.5_ exposure was associated with a 2.2% increase (95% CI: 0.9–3.5%) in psychiatric emergency department visits among older adults.^23^ The exposure studies primarily assess short-term (days to weeks) and chronic (months to years) ambient UFPs exposure. Short-term studies use lag periods of 0–7 days to capture acute effects on symptom exacerbation or emergency visits. Chronic exposure studies typically average pollution levels over 1–5 years preceding outcome assessment. Most rely on residential address-based exposure models, though recent studies incorporate time-activity patterns. Khan et al. used a comprehensive approach, including PM_2.5_, PM_10_, and NO_2_ averaged over multiple years, and found associations with multiple psychiatric disorders.^89^ Nobile et al. assessed long-term exposure (10-year average) to air pollution and incident mental disorders in a Roman cohort (N = 1.7 million), demonstrating dose–response relationships.^90^

##### Neuroinflammation in Depression

In depression, elevated inflammatory markers, including C-reactive protein (CRP) and pro-inflammatory cytokines, correlate with symptom severity and treatment resistance. The observation that brain-derived neurotrophic factor (BDNF) and CRP demonstrate dynamic, at times inverse, associations in the context of neuroimmune disturbance suggests dysregulation of neuroinflammatory and neuroplastic processes underlying environmentally mediated psychiatric disease.^91–93^

Among the most consistent findings are the associations between airborne UFPs and increased neuroinflammatory activity as evidenced by elevated systemic markers of inflammation and oxidative stress in exposed individuals.^94,95^ In parallel, alterations in circulating microRNAs and neurotrophic factors, notably BDNF, have been linked to cognitive dysfunction and psychiatric vulnerability.^96^

##### BDNF and Neuroplasticity Dysregulation

Although divergent BDNF level findings have been reported across studies, these inconsistencies likely reflect differences in particle composition, population characteristics, environmental context, and analytical methodology. BDNF plays a pivotal role in synaptic plasticity, learning, and memory, and exists in multiple biologically active isoforms, exerting region- and context-dependent effects through distinct receptor pathways.^97,98^ Thus, observed variations may be biologically meaningful rather than contradictory, highlighting complexity in interpreting neurotrophic markers across diverse exposure settings.

##### Dopaminergic Dysfunction in Mood Disorders

Critically, experimental evidence from Ji et al. (2024) demonstrates that rodent PM_2.5_ exposure decreases dopamine receptors expression in key mood regulation brain areas such as the nucleus accumbens and prefrontal cortex, mediating anxiety- and depression-like behaviors.^99^ These effects’ regional specificity—with decreased mesolimbic and mesocortical pathways receptor expression—suggests environmental nanoparticles could exacerbate the pre-existing circuit imbalances characterizing schizophrenia and depression. If human studies confirm this process, it would be highly relevant because dopaminergic dysfunction is implicated in mood disorders throughout the life cycle. Unfortunately, this study was conducted only in adult male rats.

##### Sex-Specific Psychiatric Responses

Furthermore, depression-like behavior in rodents exposed to aluminum-based nanoparticles has been linked to sex-specific stress responses, possibly driven by differential modulation of glutamate transmission and neuroendocrine dysregulation.^100^ These findings reinforce the environmentally induced psychiatric phenotype’s biological plausibility, especially when exposure occurs during critical brain development periods.

Sexual dimorphism in responses to nanopollutant exposure parallels known sex differences in psychiatric disorder prevalence and presentation. Sexual dimorphism in HPA axis activity is consistently observed in humans: women show greater stress-induced HPA axis activity variability than men, and circulating cortisol levels change across the menstrual cycle, with low levels associated with high estrogen states.^101^ Because reproductive-age women are twice as likely as men to develop stress-related psychopathologies, including depression and PTSD, cyclic estradiol fluctuations may contribute to increased female vulnerability. As previously indicated, sex-specific nanopollutant exposure has been observed, with well-differentiated male and female alterations, suggesting a link among nanopollutant interactions and pre-existing neurobiological vulnerabilities.^72,100,102^

##### HPA Axis Dysregulation in Depression

The HPA axis has been implicated in the pathophysiology of various mood and cognitive disorders. Neuroendocrine studies demonstrate HPA axis overactivity in major depression, where significant percentages of depressed patients have increased cortisol levels in saliva, plasma, and urine, and increased pituitary and adrenal gland size (and activity).^103^ This increased HPA axis activity is thought to relate, at least in part, to reduced endogenous glucocorticoid feedback inhibition mediated by the mineralocorticoid receptor (MR) and the glucocorticoid receptor (GR) binding. The relation between depression and impaired GR function seems paradoxical, since GR signaling mediates many detrimental effects associated with high cortisol levels, such as hippocampal atrophy. One explanation is that impaired GR feedback prevents the HPA axis from terminating stress responses on hour time scales, resulting in excessive cortisol levels.^104^

##### Nanopollutant-Induced HPA Axis Activation

Recent experimental evidence has shown that PM and ozone can activate the HPA axis and release glucocorticoid stress hormones (cortisol in humans, corticosterone in rodents) as part of neuroendocrine stress responses. Chronic HPA axis activation and dysregulation are associated with various adverse effects, including neurotoxicity, sensitization to other contaminants, oxidative stress, and impaired control of the inflammatory response.^105^

TNF-α can stimulate the HPA axis, leading to increased adrenal gland cortisol production and release. Several animal studies show changes in cortisol and related hormones following short-term exposure to PM_2.5_, suggesting HPA axis activation.^106^ This inflammatory pathway represents a mechanistic link between nanopollutant exposure, HPA dysregulation, and neuroinflammation—processes implicated across multiple psychiatric disorders.

##### TLR4/NF-κB-Endocrine Integration

The TLR4/NF-κB pathway serves as a critical integration point between immune activation and endocrine dysregulation. Microglial TLR4 activation leads not only to local neuroinflammation but also to systemic pro-inflammatory cytokine (TNF-α, IL-1β, IL-6) release that can cross the BBB and stimulate central HPA axis activity.^107^ This bidirectional communication between peripheral immune activation and central neuroendocrine responses creates a feed-forward cycle wherein nanopollutant-induced peripheral inflammation drives HPA axis hyperactivity, which in turn exacerbates neuroinflammation through glucocorticoid receptor dysfunction.^108,109^

##### Adolescent Vulnerability

In adolescent girls, higher PM_2.5_ concentrations predicted greater HPA-axis responsivity to social stress, with the strongest association for adolescents with more severe anxiety symptoms.^110^ The sex hormone, HPA axis regulation, and nanopollutant exposure interaction represents an understudied area potentially explaining some psychiatric disorder epidemiology sex differences.

##### Oxidative Stress in Depression and Schizophrenia

Both schizophrenia and depression involve oxidative stress and mitochondrial dysfunction—processes dramatically amplified by nanoparticle exposure. Metallic nanoparticles trigger Fenton-type reactions, causing free-radical-mediated toxicity, with increased hydrogen peroxide potential generating highly reactive hydroxyl radicals. In a recent study, PM_2.5_ exposure in a rat model induced depressive-like behaviors linked to oxidative stress and endoplasmic reticulum stress.^111^

##### Glutamatergic Dysfunction in Schizophrenia

In schizophrenia, neuroinflammation interacts with glutamatergic and dopaminergic dysfunction to produce cognitive deficits and positive symptoms. The microglia hypothesis of schizophrenia proposes that aberrant microglial activation contributes to synaptic pruning abnormalities during neurodevelopment—a process that nanopollutant-induced neuroinflammation could exacerbate, particularly during critical developmental windows. Nanopollutant-induced glutamatergic neurotransmission disruption may represent a mechanism through which environmental exposures interact with glutamate receptor genetic vulnerabilities (particularly, NMDA receptor-related genes) to increase schizophrenia risk.

##### Cognitive Effects Across Lifespan

Cognitive impairments linked to nanopollutant exposure have been demonstrated in animal models. Experimental data show UFPs impair short-term memory, learning, and executive function in sex- and dose-dependent manners. Specifically, males display heightened vulnerability in memory-related tasks, while females exhibit vulnerability in motivational domains.^28,112^ These behavioral alterations correlate with brain morphology, gene expression, and glutamatergic signaling pathway changes.^15,113^

##### Integrated Pathophysiological Model

TLR4/NF-κB-mediated neuroinflammation, HPA axis dysregulation, and neurotransmitter dysfunction convergence creates a pathophysiological triad underlying environmentally induced psychiatric disorders.^114^ Nanopollutant exposure activates peripheral and central TLR4, triggering NF-κB-dependent pro-inflammatory cytokine production. These cytokines stimulate the HPA axis while simultaneously promoting microglial activation and aberrant synaptic pruning.^115^ Chronic HPA activation leads to glucocorticoid resistance, impairing normal inflammatory response termination. Meanwhile, excessive complement-mediated synaptic elimination disrupts dopaminergic and glutamatergic circuits critical for mood regulation and cognition.

This integrated model explains how environmental nanopollutants can produce disorder-specific psychiatric phenotypes depending on which triad component is most affected: HPA hyperactivity with glucocorticoid resistance predominates in depression; dopaminergic circuit dysfunction combined with aberrant synaptic pruning characterizes schizophrenia; and dysregulated neurodevelopmental synaptic refinement contributes to ASD.

##### Epidemiological Support

Population-based studies further support the psychiatric relevance of environmental nanopollutants. Longitudinal data have linked chronic UFP exposure to increased anxiety, depression, schizophrenia, and neurodevelopmental disorder risk.^115,116^

Three primary pathways link UFP exposure to psychiatric disorders: (1) *Neuroinflammation*: UFP-induced microglial activation and pro-inflammatory cytokine release (IL-6, TNF-α, IL-1β) affect neurotransmitter metabolism and synaptic plasticity. Kilian and Kitazawa (2018) review evidence that systemic inflammation from air pollution exposure activates brain inflammatory cascades.^117^ (2) *HPA-axis dysregulation*: UFPs trigger hypothalamic-pituitary-adrenal axis hyperactivity, leading to elevated cortisol and stress hormone dysregulation implicated in depression. Thomson proposed an allostatic load model in which chronic pollution exposure disrupts stress response systems.^105^ (3) *Neurotransmitter disruption*: Dopaminergic and serotonergic pathways are particularly vulnerable. Ji et al. demonstrated that PM_2.5_ exposure interferes with dopamine receptors via phenyl-containing compounds, inducing anxiety- and depression-like behaviors in mice.^99^ Kim et al. showed PM_2.5_ exposure triggers hypothalamic oxidative stress and ER stress, leading to depressive-like behaviors via HPA-axis activation.^111^

There are some key limitations, as many studies rely on self-reported depressive symptoms or anxiety scales rather than clinical diagnoses, introducing measurement error. Also, inadequate control of lifestyle factors (physical activity, smoking, alcohol consumption, diet, and social isolation) is inconsistently measured and may result in incorrect associations between pollution and mental health. In the same way, studies finding positive associations are more likely to be published than those reporting null findings. Time-series studies capture acute symptom exacerbation but cannot assess whether pollution contributes to chronic disease development.

#### (iii) Neurodegenerative Disorders (Late-Life Outcomes)

Progressive neurological conditions, including Alzheimer’s disease (AD), Parkinson’s disease (PD), amyotrophic lateral sclerosis (ALS), and age-related cognitive decline characterized by irreversible neuronal loss and functional impairment. A moderate to strong association is emerging from long-term cohort studies. For Alzheimer’s disease/dementia, Lee et al. (2019) reported HR = 1.049 (95% CI: 1.048–1.051) for dementia-related hospitalizations per 1 μg/m^3^ PM_2.5_ increase among Medicare beneficiaries (N = 13.3 million).^118^ Wu et al. (2022) found a 10 μg/m^3^ increase in NO_2_ associated with a 19% higher dementia risk (HR = 1.18, 95% CI: 1.04–1.33) in a Swedish cohort.^119^ For Parkinson’s disease, a meta-analysis by Kasdagli et al. (2018) estimated pooled RR = 1.03 (95% CI: 1.01–1.05) per 10 μg/m^3^ PM_2.5_.^120^ Specifically for UFPs, Yu et al. demonstrated long-term UFPs exposure associated with ALS incidence in The Netherlands (HR = 1.09 per IQR, 95% CI: 1.02–1.17).^121^ Weichenthal et al. found within-city UFPs concentration variations associated with brain tumor incidence (HR = 1.10 per IQR, 95% CI: 1.02–1.19).^122^

Studies assess lifetime cumulative exposure or late-life chronic exposure (typically 10–30 years). Exposure reconstruction uses historical residential addresses linked to spatiotemporal pollution models. For example, Power et al. used black carbon as a UFPs tracer over 10-year periods in the Nurses’ Health Study, finding associations with cognitive decline equivalent to 1–2 years of brain aging.^123^ Chen et al. (2017) demonstrated that decades-long exposure to traffic-related air pollution increased dementia incidence in Ontario (N = 6.6 million).^124^ Recent studies incorporate source-specific exposure metrics: Mussalo et al. showed traffic-related UFPs impair mitochondrial function in human olfactory mucosa cells, providing direct mechanistic evidence linking UFPs exposure to AD-relevant pathology.^125^

##### Nanoparticle-Induced Protein Misfolding

Among the most concerning mechanisms is the nanoparticle’s potential to initiate or accelerate protein aggregation—a hallmark of multiple neurodegenerative conditions. Nanoparticles can promote nucleation and misfolding of amyloidogenic proteins, including Aβ and α-synuclein, depending on their size, surface charge, and physicochemical properties.^126–130^ These interactions may destabilize neuronal membranes and initiate cascades that lead to synaptic loss and neurodegeneration.

##### TLR4-Mediated Protein Aggregate Clearance

TLR4 on microglia surfaces participates in α-synuclein phagocytosis, with TLR4 ablation impairing phagocytosis and TLR4 absence leading to inflammasome inhibition.^131^ Endogenous α-synuclein can activate microglia by binding to TLR2, but this activation is conformation-dependent—only the oligomeric forms, not monomeric or dimeric forms, act as TLR2 agonists capable of triggering inflammatory factor release.^132^

Autophagy plays a crucial role in glial protein aggregate degradation, with microglia internalizing neuron-released α-synuclein and sequestering it within autophagosomes for lysosomal degradation—a process regulated by TLR4-NF-κB signaling, which induces p62 expression, an adaptor protein that binds α-synuclein and facilitates its recruitment to autophagosome.^133^ In TLR4 absence, p62 upregulation is disrupted, impairing α-synuclein delivery to microglial lysosomes, and autophagy-deficient microglia fail to clear α-synuclein, resulting in its accumulation and subsequent neuronal death.^134^

##### Alzheimer’s Disease Pathology

Evidence suggests that UFPs may contribute to neurodegenerative disease pathology, with repeated or sustained exposure modifying tau phosphorylation and altering microglial morphology.^1,14,62^ Specific nanoparticle types, including aluminum oxide and silica-based particles, have been associated with increased deposition of AD-related protein, particularly in vulnerable brain regions such as the hippocampus and cerebellum.^135^

##### Mitochondrial Dysfunction in AD

Air pollution exposure involves complex copollutant mixtures acting synergistically to exacerbate neurotoxic outcomes. Within the BREATH initiative, evidence shows coexposure to NO_2_, PAHs, and PM—especially UFPs—acts synergistically to drive oxidative stress, neuroinflammation, and progressive neurodegeneration.^136^ UFPs chemical composition matters: transition metal-, quinone-, or persistent organic-rich particles have higher oxidative potential and greater capacity to disrupt BBB integrity and activate microglia, whereas inert mineral UFPs exert weaker neurotoxic signals.^137^ UFPs can translocate to brain tissue, providing a direct vehicle for these surface-bound chemicals to trigger sustained neuroinflammation and proteinopathy pathways linked to AD and PD.^125^ This interplay underscores the importance of considering pollutant mixtures rather than isolated compounds in assessing neurotoxic risks.

##### Chronic Neuroinflammation and Oxidative Damage

Preclinical studies demonstrate that nanopollutants, particularly UFPs, impact the brain through multiple converging mechanisms: neuroinflammation, oxidative stress, altered neurotransmission, and disruption of brain structure and function. These effects may contribute to neurodevelopmental or neurodegenerative trajectories depending on exposure timing and duration.^138–142^ Mechanistically, UFPs induce peripheral and central inflammation by activating immune pathways and releasing proinflammatory cytokines.^143,144^ Evidence indicates they generate ROS, which disrupt cellular homeostasis, leading to lipid peroxidation, DNA damage, and impaired neuronal signaling.^145,146^ These effects are not limited to specific cell types but involve astrocytic and microglial activation, contributing to a chronic proinflammatory milieu in the brain.

Despite substantial preclinical evidence linking combustion-derived ultrafine particle (UFP) exposure to neuroinflammation, oxidative stress, and behavioral deficits, human data remain limited. This limitation partly reflects the inherent ethical and logistical challenges of studying neurological outcomes under controlled exposure conditions. Nevertheless, emerging epidemiological, clinical, and biomarker-based investigations are beginning to clarify mechanisms and brain-related outcomes relevant to environmental nanoparticle exposures. Controlled human exposure studies provide important translational insights; however, it is critical to recognize that many such studies (e.g., diesel exhaust, cooking aerosols, gas stove emissions) involve complex pollutant mixtures. Although ultrafine particles constitute a substantial fraction of these emissions by number concentration, causal attribution to nanoparticles *per se* is constrained by concurrent exposure to gaseous pollutants and organic compounds.

##### Excessive Synaptic Pruning in Neurodegeneration

Excessive adult brain synaptic pruning can be detrimental and has been associated with synaptic loss in several pathological conditions.^77^ Aged C3-deficient mice show reduced age-dependent synapse and neuronal loss in hippocampal CA3, together with enhanced long-term potentiation and cognition.^78^

##### Proteinopathy-Psychiatric Disorder Continuum

Growing literature is beginning to link proteinopathies not only to neurodegeneration but also to psychiatric disorders. Recent studies have proposed mechanistic overlaps between early protein misfolding and the pathogenesis of schizophrenia and mood disorders.^147,148^ Concurrently, brainstem structure and function abnormalities have been implicated in ASD pathophysiology,^149^ further supporting the brainstem as an early environmental damage target.

This emerging understanding challenges the traditional dichotomy between “neurodegenerative” and “psychiatric” disorders, suggesting instead a continuum of protein aggregation-related pathophysiology manifesting differently depending on affected brain regions, exposure timing, and genetic vulnerability.

##### Metal Effects on Neurodegeneration

Potter et al. (2021) emphasize that early exposure may increase adult depression risk by causing persistent neuroinflammation, oxidative stress, and protein dysfunction, with PM-bound Mn associated with impaired motor skills in older adults.^84^

##### ALS and Brain Tumor Associations

Associations have also been reported between ambient PM and neurological disease progression, amyotrophic lateral sclerosis (ALS), where chronic UFPs exposure appears to increase susceptibility.^121^ Additional studies have reported elevated malignant brain tumor incidence.^122^

##### Neuropathological and Imaging Evidence

Imaging and neuropathological study findings corroborate these epidemiological trends. Notably, air pollutant exposure has been associated with structural brain changes such as cortical thinning and altered white matter integrity, and increased cerebrospinal fluid TDP-43 levels—a hallmark of several neurodegenerative diseases.^63^

#### (iv) Cross-Cutting Mechanisms

Four interconnected pathways drive neurodegeneration: (1) *Chronic neuroinflammation*: Persistent microglial activation and astrogliosis create pro-inflammatory brain environments. Calderón-Garcidueñas et al. (2008) demonstrate that long-term exposure to air pollution sustains neuroinflammatory states conducive to protein misfolding.^150^ (2) *Oxidative damage accumulation*: Decades of UFP-induced reactive oxygen species production damage mitochondrial DNA, proteins, and lipids. Mussalo et al. (2024) showed that traffic UFPs impair mitochondrial respiration in human olfactory cells, consistent with mitochondrial dysfunction in AD.^125^ (3) *Amyloid-β aggregation*: Nanoparticles may act as nucleation sites for amyloid plaque formation.^126^ Herr et al. (2021) demonstrated that concentrated ambient UFPs accelerate amyloid pathology in transgenic AD mice.^135^ (4) *Tau phosphorylation*: Maher et al. (2016) found magnetite nanoparticles in human brain tissue colocalized with tau tangles and amyloid plaques, suggesting direct nanoparticle involvement in AD pathology.^3^

Some key limitations of these studies are that individuals who survive to old age may be systematically different (healthier, less pollution-sensitive) from those who die earlier. potentially attenuating associations. Also, cardiovascular mortality from air pollution may preclude neurodegenerative disease diagnosis, leading to an underestimation of neurological effects. It is also difficult to reconstruct historical exposures, as many participants may have moved multiple times over decades, so residential address history may be incomplete. Temporal changes in emission sources (e.g., the introduction of catalytic converters, a shift from coal to natural gas heating) alter the composition of pollution, complicating exposure assessment. Finally, as neurodegenerative diseases result from complex interactions among genetics (APOE ε4, LRRK2), lifestyle factors, comorbidities, and environmental exposures, isolating air pollution’s contribution is challenging.

Several exposure effects cannot be associated with a single specific category. They include, for example, the TLR4/NF-κB inflammatory cascade. Toll-like receptor 4 (TLR4) is essential for microglial responses against pathological proteins *in vitro*, including protein uptake, pro-inflammatory cytokine release, and ROS production.^151^ The TLR4/NF-κB pathway represents a critical mechanistic link between nanopollutant exposure and neuroinflammation. Injury-mediated microglial activation transiently upregulates TLR4 protein, and NF-κB inhibition suppresses expression of TLR4 and downstream genes, including protein P50, IL-6, and TNF-α.^152^

This cascade—wherein TLR4 activation leads to MyD88-dependent IKK activation, subsequent IκB-α phosphorylation and degradation, and finally nuclear translocation of NF-κB p65—orchestrates transcription of pro-inflammatory genes including TNF-α, IL-1β, IL-6, iNOS, and COX-2.^153^

Also, microglial activation and neuronal dysfunction occur, as chronic activation via bacterial lipopolysaccharide through TLR4 or IFN-γ induces reactive microglial phenotypes associated with morphological changes, population expansion, upregulation of CD11b and CD68, and proinflammatory cytokine (IL-1β, TNF-α, IL-6) and NO release. Notably, TLR4 and IFN-γ receptor coactivation results in neuronal dysfunction and death, mainly due to enhanced microglial iNOS expression and NO release.^154^ BBB translocation and direct neurotoxicity also play an important role, as UFPs can cross the BBB and enter the brain within 4 to 24 h after inhalation, accumulating and exerting direct neurotoxic effects.^139,143^ Mechanistic studies highlight specific nanopollutant entry pathways into the brain, including endocytosis, transcytosis, and olfactory nerve transport, emphasizing roles of particle composition (metallic components, black carbon, organic fractions) and copollutants such as NO_2_ and hydrocarbons in modulating neurotoxic effects.^155^ There is also human evidence from controlled exposure studies demonstrating translational relevance: fMRI connectivity changes following diesel exhaust exposure,^156^ EEG alterations after controlled diesel exhaust inhalation,^157^ altered brain activity following exposure to frying aerosols,^158^ and nervous system responses to gas stove emissions.^159^ These studies provide direct functional evidence linking UFPs exposure to acute changes in brain physiology.

It has been reported that nanoparticle deposition leads to the formation of molecular oxygen-dependent superoxide anion radicals, hydrogen peroxide, and hydroxyl radicals, producing inflammatory phagocyte release. NO_2_ and PAHs increase the formation of nitrogen/oxygen species and the bioactivation of toxic intermediates. Simultaneously, UFPs’ large surface area concentrates redox-active metals and organic toxic substances, amplifying ROS production, mitochondrial damage, and neural tissue lipid peroxidation.^160^ The exposure timing, duration, and developmental stage determine which pathophysiological processes dominate and which psychiatric outcomes emerge. Repeated stressor exposure or sustained stress periods can result in HPA axis dysregulation and altered immune and inflammatory responses. Air pollution exposure, even at low doses, occurs daily, impacts accumulate over time, and may have effects similar to chronic stress. Finally, together, these findings signal the need for a paradigm shift in understanding and managing psychiatric and neurological diseases. Environmental nanopollutants—particularly UFPs—represent an underappreciated, modifiable risk factor. Clinicians, neuroscientists, and public health authorities must prioritize strategies that reduce exposure, improve surveillance, and integrate environmental history into mental health assessment and prevention frameworks. Public health recommendations further emphasize limiting indoor combustion sources and protecting vulnerable groups, particularly pregnant women, children, and adults.^82,83,121^

Table 1 provides detailed findings from reviewed studies, including study type (animal, human, or in vitro) for clarity.

**Table 1:** Summary of Studies Evaluating Neuropsychiatric and Neurodevelopmental Effects of UFPs and Environmental Nanoparticles

Reference	Exposure Type	Model/Population	Neurological/Psychiatric Outcome	Key Findings	Study Type
^72^	Metal nanoparticles	In vitro neurons	Synaptic function, oxidative stress	Altered neurotransmission, elevated oxidative markers	**In vitro**
^100,112^	ENPs, UFPs	Rodent models	Cognitive and motivational domains	Sex-specific effects: male-biased memory deficits, female-biased motivational alterations	**Animal**
^72,79^	Prenatal exposure to UFPs	Birth cohorts	Cortical thickness, ASD risk	Reduced cortical thickness; increased autism risk	**Human**
^156^	Diesel exhaust	Controlled human exposure (fMRI)	Functional connectivity	Altered brain connectivity after exposure	**Human**
^157^	Diesel exhaust	Controlled human exposure (EEG)	Electrophysiological activity	Acute EEG changes following exposure	**Human**
^158^	Frying aerosols	Controlled human exposure	Brain activity	Altered activity patterns postexposure	**Human**
^159^	Gas stove emissions	Controlled human exposure	Nervous system response	Physiological and neurological responses to cooking emissions	**Human**
^94,95^	Diesel exhaust particles	Rodent models	Neuroinflammation, protein aggregation	Oxidative stress, mitochondrial dysfunction, protein aggregation	**Animal**
^90,121^	Ambient nanopollutants	Adults	Depression, anxiety, schizophrenia, ALS	Higher prevalence of psychiatric and neurodegenerative disorders	**Human**
^63,70^	Urban air pollution (metal-rich nanoparticles)	Autopsy brains (Mexico City residents)	Neuropathology: tau, TDP-43, α-synuclein	Nanoparticles colocalized with protein aggregates and neuroinflammation	**Human**
^82,83^	Traffic-related air pollution	Schoolchildren	Cognition, working memory, attention	Slower cognitive development with chronic exposure	**Human**

### Human Evidence in the Pediatric Population: a Unique Window of Vulnerability

The developing brain is uniquely vulnerable to environmental damage. During the prenatal and early postnatal periods, rapid neuronal proliferation, migration, and synaptogenesis create a landscape of heightened plasticity—but also increased susceptibility to disruption. Affectations during these critical windows can have long-lasting and, in some cases, irreversible effects on behavior and mental health.^39^ Neurodevelopmental plasticity, while often beneficial, may become maladaptive under chronic or high-dose nanopollutant exposure. The same flexibility enabling recovery in enriched environments can exacerbate risk in toxic ones, particularly when exposures are sustained or cumulative.^161,162^ For these reasons, pediatric population data must be interpreted separately from adult studies.

Mechanistic insights further illustrate this vulnerability. UFPs can reach the pediatric brain through multiple routes: endocytosis, BBB transcytosis, and olfactory nerve transport. Their effects are shaped by particle composition, including metallic constituents, black carbon, and organic fractions, which may enhance neurotoxicity. Moreover, copollutants such as NO_2_ and hydrocarbons act synergistically, exacerbating oxidative stress, neuroinflammation, and structural disruption during critical periods of brain development.

### General Limitations in Human Nanotoxicology Research

A central limitation in studying nanopollutants and their effects on human brain health is the ethical infeasibility of experimental exposure designs. Unlike pharmacological trials, randomized controlled airborne nanoparticle exposures are not permissible in human subjects due to potential harm. Consequently, the existing evidence base remains predominantly observational, relying on retrospective exposure models, epidemiological associations, and preclinical data to infer neurotoxic risk.

One of the most significant technical challenges is quantifying and characterizing airborne nanoparticles. For much of the 20th century, UFPs were not considered discrete environmental pollutant nanoparticles. Technological advances have enabled ambient air particle detection and monitoring only in recent decades. Previously, nanopollutants remained undetectable, and historical exposure reconstructions relied on indirect proxies such as land use, traffic density, and particulate composition. Each research group has developed unique modeling strategies to estimate historical nanoparticle levels across space and time, but these methods vary widely and are difficult to compare directly. This methodological heterogeneity contributes to exposure-response relationship uncertainty and limits result reproducibility and comparability across cohorts.^85,149^

Compounding this issue is inherent literature geographic bias: many large-scale epidemiological studies have been conducted in high-income countries with relatively low ambient pollution background levels. As a result, their findings may not apply to low- and middle-income country populations, where pollutant concentrations are often higher and more complex. Such disparity underscores urgent need for inclusive, global research frameworks and more representative data sets.^163^ Standardization of exposure assessment techniques, including those used to measure nanoparticle size, concentration, chemical composition, and surface characteristics, is also lacking. The absence of harmonized protocols hampers meta-analyses and the derivation of clinically meaningful thresholds for neurotoxic exposure. Moreover, current research methodologies rarely account for the coexistence of multiple pollutants, allergens, or psychosocial stressors. These elements may interact synergistically with nanoparticles, intensifying their impact on neural and mental health outcomes—a complexity that most observational designs are currently unable to disentangle.^163,164^

To advance the field, a coordinated, multidisciplinary approach is essential. This requires integrating environmental science, exposure biology, neuroscience, and epidemiology while developing ethical frameworks that enable critical research while protecting vulnerable populations. Establishing a global monitoring system for airborne nanopollutants—including real-time data on concentration, composition, and geographic distribution—will be pivotal for effective risk assessment and public health response. Public health strategies should further emphasize limiting indoor combustion sources and safeguarding vulnerable groups, particularly pregnant women and children, as part of comprehensive efforts to prevent exposure.

### Mechanistic Studies and Clinical Themes

Particulate matter exposure has been positively associated with ASD and schizophrenia diagnosis rates. Mechanistic studies demonstrate that PM exposure leads to brain inflammation and white matter pathologies consistent with these disorders.^165^ PM exposure has been associated with neurological abnormalities affecting neurodevelopment, neuroplasticity, and behavior, with growing interest in investigating possible relationships between PM exposure and neurodegenerative disease onset and progression, including AD, PD, Huntington’s disease, and multiple sclerosis.^166^

For early life exposures, the evidence derives predominantly from observational cohort studies with prospective exposure assessment. While causality cannot be definitively established, temporal relationships—prenatal/early life exposure preceding diagnosis by years—strengthen causal inference. Large population-based cohorts provide robust statistical power: the Danish National Birth Cohort (Lim et al., 2024; N = 398,063)^167^ and California-based studies (Volk et al., 2013) demonstrate consistency across populations.^71^ However, residual confounding remains possible despite adjustment for maternal education, socioeconomic status, and prenatal factors. While for psychiatric/mood disorders across the lifespan, the evidence of cross-sectional and time-series studies dominates the psychiatric disorder literature, limiting causal inference. Directionality remains uncertain: does pollution cause depression, or do depressed individuals have different exposure patterns? Reverse causation is plausible—individuals with mental illness may spend more time indoors near high-traffic areas, alter physical activity patterns, or relocate to more polluted neighborhoods due to socioeconomic factors. Longitudinal cohort studies with repeated outcome assessments (e.g., Yang et al., 2023; Nobile et al., 2023) provide stronger evidence by adjusting for baseline mental health status, but cannot fully exclude bidirectional relationships.^25,90^ Controlled exposure studies in humans (Gawryluk et al., 2023) demonstrate acute neurological effects of diesel exhaust, supporting biological plausibility.^156^ Finally, for late-life outcomes, evidence from long-term prospective cohort studies with decades-long follow-up provides the strongest evidence, though causality remains suggestive rather than conclusive. Studies benefit from large sample sizes (often >100,000 participants) and clinical outcome ascertainment through medical registries. However, long latency periods (20–40 years between exposure initiation and clinical diagnosis) complicate exposure-outcome linkage. Historical exposure reconstruction relies on models that inherently introduce uncertainty. Triangulation across study designs strengthens inference: epidemiological associations + neuropathological findings + controlled animal experiments + human biomarker studies collectively support causal pathways.^81,125,168^

Overlapping neuropathology in AD, PD, frontotemporal lobar degeneration, and ALS begins in the first two decades of life in pollution-exposed urban residents, with brain UFPs and industrial nanoparticles serving as key players.^169^ Children are especially susceptible to these effects due to their rapidly developing nervous systems.^170^ Females are more vulnerable than males to air pollution mental health effects, with women showing stronger associations between perceived air pollution and poor health.^171^ Among 41,844 middle-aged and older women in the Nurses’ Health Study, long-term PM_2.5_ and ozone exposure were associated with depression onset.^172^ Recent 2025 reviews demonstrate that air pollution impacts span ASD, ADHD, AD, and PD through multiple pathways including BBB disruption, oxidative stress, and neuroinflammation, establishing PM as a critical modifiable risk factor across the lifespan with sex-specific vulnerabilities requiring targeted public health interventions.^173^

The evidence synthesized in this review demonstrates associations between UFPs and nanoparticle exposure and neurological outcomes, but does not establish causation. Overall, regarding neurodevelopmental disorders, there is evidence suggesting a causal relationship, but not definitive. A temporal sequence is favorable, but residual confounding cannot be excluded. For psychiatric disorders, the evidence indicates an association, but causality remains uncertain. Reverse causation, confounding, and lack of experimental evidence are major limitations. Finally, on neurodegenerative disorders, the evidence is suggestive but insufficient for causal inference. Long latency, survival bias, and exposure misclassification weaken causal claims.

## Conclusion and Future Directions

Cumulative evidence from human and experimental studies increasingly supports associations between exposure to UFPs and metal-rich environmental nanopollutants and adverse neurological and psychiatric outcomes. These include cognitive impairment, neurodevelopmental disorders such as ASD, altered stress reactivity, and elevated incidence of both mental illness and neurodegenerative conditions. Further research is needed to understand the specific relationship between nanopollutants and the physiological mechanisms by which they achieve their multiple effects. There is still sparse literature on this topic, which needs to be critically reviewed, including studies with human subjects that consider sociocultural group variations and subjects with different exposures. This systematic review synthesizes evidence suggesting associations between environmental exposure to UFP and nanoparticles and adverse neurological outcomes across the lifespan. While mechanistic studies provide strong biological plausibility and animal models demonstrate neurotoxic effects, human epidemiological evidence remains predominantly observational and cannot establish causation.

Epidemiological findings highlight developing brain increased vulnerability to nanopollutants. Studies by Lubczyńska et al. (2020) and Chen et al. (2018) demonstrate associations between early exposure and reduced cortical thickness and increased autism risk, respectively, reflecting critical in utero and infancy vulnerability windows.^162,163^ School-based longitudinal studies by Forns et al. (2017) and Sunyer et al. (2015) further reveal that chronic exposure to traffic-related air pollution slows cognitive development, particularly affecting working memory and attention.^79,83^

In adult populations, chronic nanopollutant exposure has been associated with increased depression, anxiety, schizophrenia, and ALS prevalence, as shown by Nobile et al. (2023) and Yu et al. (2021).^90,121^ Neuropathological and neuroimaging studies reinforce these clinical findings. Maher et al. (2016) and Calderón-Garcidueñas et al. (2022a, 2022b) reported the presence of metallic and magnetic nanoparticles in human brain tissue, colocalized with hyperphosphorylated tau, TDP-43, and α-synuclein, along with elevated neuroinflammatory markers, suggesting plausible mechanistic links to early neurodegenerative changes.^3,84,122^

Although exact biological mechanisms are not yet fully understood, studies by Liu et al. (2017, 2018) and Calderón-Garcidueñas et al. (2022a) consistently indicate that oxidative stress, neuroinflammation, mitochondrial dysfunction, and protein aggregation are central pathways in particle-associated neurotoxicity observed in experimental and high-exposure contexts.^63,94^ Moreover, transplacental transfer has been demonstrated through advanced microscopy studies, showing that nanoparticles can reach the fetal brain, potentially with lifelong consequences.^63^ Using advanced microscopy techniques, Calderón-Garcidueñas and colleagues demonstrated that urban pollution nanoparticles can traverse the placenta and accumulate in the embryonic brain, even during early gestation.^174^ These findings raise serious concerns about potential lifelong neurological consequences following prenatal exposure, including altered neurodevelopmental trajectories and increased later-life psychiatric disorder susceptibility. These observations indicate an urgent need for a paradigm shift in assessing pediatric neurodevelopmental risk. Combustion-related ultrafine and metal-rich particles—often invisible and insufficiently regulated—may contribute to early and persistent alterations in brain structure and function, and psychiatric vulnerability particularly during sensitive developmental windows. Environmental nanopollutants—often invisible and unregulated—may act as early and persistent modifiers of brain structure, function, and psychiatric vulnerability. Clinicians should consider early environmental exposure history in developmental and behavioral assessments, and public health strategies must prioritize reducing nanopollutant exposure during critical brain development windows. In particular, limiting indoor combustion sources and protecting vulnerable groups such as pregnant women and children should be central to prevention efforts.

Although current epidemiological evidence is moderate in strength, it aligns with mechanistic data from experimental models and is consistent with broader air pollution research findings. Nanopollutant neurodevelopmental and neuropsychiatric effects, particularly from early life exposures, are increasingly recognized as plausible and preventable contributors to global disease burden. No established specific annual safe exposure standard or guideline value exists for UFPs (PM_0_._1_), despite recent scientific evidence suggesting these particles pose the greatest risk due to their increased ability to penetrate lungs, cross the BBB, and directly enter the bloodstream. The strength of evidence varies by outcome: temporal relationships are strongest for neurodevelopmental disorders (prenatal/early life exposure preceding diagnosis), weakest for psychiatric disorders (cross-sectional studies dominating), and emerging for neurodegenerative conditions (limited long-term cohort data). It is not affirmed that UFPs exposure causes these disorders, but rather that accumulating evidence requires further prospective cohort studies with improved exposure assessment (personal monitoring, source apportionment). Also, a research design that strengthens causal inference (e.g., natural experiments, sibling-comparison designs, Mendelian randomization) is desirable, as are mechanistic studies in humans (e.g., neuroimaging and biomarker studies) in order to validate animal findings. Finally, a precautionary public health approach is needed, given suggestive evidence and biological plausibility, even in the absence of definitive causal proof. The distinction between association and causation is not semantic—it fundamentally shapes appropriate public health responses and research priorities. This review supports continued investigation and precautionary measures while acknowledging substantial uncertainty regarding causality.

The socioeconomic implications are considerable. These outcomes impact not only cognitive and emotional function—with expected impacts on work productivity, school performance, mental health, and personal relationships—but also place growing burdens on healthcare systems, escalate private insurance costs, and widen psychiatric and neurological care access disparities. Without intervention, a significant proportion of the global population, particularly in resource-limited settings, may face diminished access to appropriate multidisciplinary care. Furthermore, over the past 25 years, concentrations of fine and coarse PM in the environment have increased, particularly in low- and middle-income countries, where the majority of the world’s population lives. In 1980, initial estimates indicated global average PM_2.5_ concentrations were approximately 30 μg/m^3^, while by 2000 this average had increased to 37 μg/m^3^, and is currently reaching 44 μg/m^3^ in Asia. Overall, the Gini index for global air quality increased from 0.30 in 2000 to 0.35 in 2020, indicating inequality in PM_2.5_ exposure across countries. In contrast, the World Health Organization (WHO), based on emerging evidence, now notes in its 2021 guidelines that levels above 5 μg/m^3^ for PM_2.5_ and 15 μg/m^3^ for PM_10_ pose significant health risks. In 2023, only seven countries (Australia, Estonia, Finland, Grenada, Iceland, Mauritius, and New Zealand) met WHO PM_2.5_ standards.^175^

To address this challenge, several priorities must be pursued. First, sex-specific analyses are essential, as both experimental and epidemiological evidence highlight gender differences in behavioral and cognitive nanoparticle exposure responses.^95,176^ Second, the field urgently needs standardized airborne nanoparticle measurement methods, including accurate size, concentration, and chemical composition assessments, to facilitate interstudy comparability and policy relevance. Third, integrating environmental exposure into clinical mental health assessment is important. Future research should adopt a lifespan approach, examining developmental, adult, and aging brain outcomes, with particular emphasis on prenatal exposure and potential transgenerational effects, which remain poorly understood.

Additionally, vulnerable population protection—notably pregnant women, children, and individuals with genetic or autoimmune predispositions—must become a public health priority. Public health recommendation for regulatory reform are warranted, emphasizing limiting indoor combustion sources and reducing pregnancy and childhood exposure. Although methodological heterogeneity persists, the convergence of epidemiological associations and mechanistic experimental findings supports precautionary environmental policy measures to reduce combustion-related ultrafine particle emissions, particularly in densely populated regions. Importantly, future research must adopt standardized exposure definitions and measurement frameworks that distinguish size-defined UFPs, composition-specific ambient nanoparticles, and engineered nanomaterials, thereby improving causal inference and regulatory applicability.

Our recommendations align with recent calls from both the US EPA and the WHO for enhanced research on UFPs and potential regulatory development. The EPA’s 2022–2026 Strategic Research Action Plan identifies particle number concentration as a priority research area,^176^ while WHO’s 2023 technical report on UFPs emphasizes the need for neurodevelopmental outcome studies.^41^ By demonstrating consistent associations between UFPs exposure and mental health burdens across multiple study designs and populations, this review provides evidence to support the integration of neuropsychiatric end points and PNC-based metrics into established regulatory frameworks, thereby enhancing their protective capacity for public health.

In summary, there is an urgent need to recognize environmental nanopollutants as a modifiable risk factor for mental and neurological disorders. Integrating exposure history into clinical practice, advancing multidisciplinary research, and enacting forward-looking public health policies are essential steps to mitigate long-term neurological consequences of this largely invisible yet pervasive environmental threat.

## ⊥

Facultad de Psicología, Benemérita Universidad Autónoma de Puebla, 3 Oriente 403, Centro Histórico, Puebla, 72000 Puebla, México
